# Effects of co-administration of candesartan with pioglitazone on inflammatory parameters in hypertensive patients with type 2 diabetes mellitus: a preliminary report

**DOI:** 10.1186/1475-2840-12-71

**Published:** 2013-05-02

**Authors:** Hirofumi Suzuki, Masaya Sakamoto, Takeshi Hayashi, Hiroyuki Iuchi, Kennosuke Ohashi, Tsuyoshi Isaka, Noriko Sakamoto, Yosuke Kayama, Katsuyoshi Tojo, Michihiro Yoshimura, Kazunori Utsunomiya

**Affiliations:** 1 Department of Internal Medicine, Division of Diabetes, Metabolism and Endocrinology, Jikei University School of Medicine, 3-25-8 Nishi-Shinbashi, Minato-ku, Tokyo 105-8461, Japan; 2 Department of Internal Medicine, Division of Cardiology, Jikei University School of Medicine, 3-25-8 Nishi-Shinbashi, Minato-ku, Tokyo 105-8461, Japan

**Keywords:** Candesartan, Angiotensin receptor blockers, Type 2 diabetes mellitus, Inflammatory parameters, Pulse pressure

## Abstract

**Background:**

Angiotensin receptor blockers (ARBs) are reported to provide direct protection to many organs by controlling inflammation and decreasing oxidant stress. Pioglitazone, an anti-diabetic agent that improves insulin resistance, was also reported to decrease inflammation and protect against atherosclerosis. This study aimed to evaluate the utility of combination therapy with both medicines from the viewpoint of anti-inflammatory effects.

**Methods:**

We administered candesartan (12 mg daily) and pioglitazone (15 mg daily) simultaneously for 6 months to hypertensive patients with type 2 diabetes mellitus (T2DM) and evaluated whether there were improvements in the serum inflammatory parameters of high-molecular-weight adiponectin (HMW-ADN), plasminogen activator inhibitor-1 (PAI-1), highly sensitive C-reactive protein (Hs-CRP), vascular cell adhesion molecule-1 (VCAM-1), and urinary-8-hydroxydeoxyguanosine (U-8-OHdG). We then analyzed the relationship between the degree of reductions in blood pressure and HbA1c values and improvements in inflammatory factors. Furthermore, we analyzed the relationship between pulse pressure and the degree of lowering of HbA1c and improvements in inflammatory factors. Finally, we examined predictive factors in patients who received benefits from the co-administration of candesartan with pioglitazone from the viewpoint of inflammatory factors.

**Results:**

After 6 months of treatment, in all patients significant improvements from baseline values were observed in HMW-ADN and PAI-1 but not in VCAM-1, Hs-CRP, and U-8-OHdG. Changes in HbA1c were significantly correlated with changes in HMW-ADN and PAI-1 in all patients, but changes in blood pressure were not correlated with any of the parameters examined. Correlation and multilinear regression analyses were performed to determine which factors could best predict changes in HbA1c. Interestingly, we found a significant positive correlation of pulse pressure values at baseline with changes in HbA1c.

**Conclusions:**

Our data suggest that the pulse pressure value at baseline is a key predictive factor of changes in HbA1c. Co-administration of candesartan with pioglitazone, which have anti-inflammatory (changes in HMW-ADN and PAI-1) effects and protective effects on organs, could be an effective therapeutic strategy for treating hypertensive patients with type 2 diabetes mellitus.

**Trial registration:**

UMIN-CTR: UMIN000010142

## Background

It is well established that hypertension complicated with T2DM results in an increased incidence of cardiovascular disease [1]. Hence, to reduce cardiovascular events in patients with T2DM, treatment of hypertension in addition to glycemic control is important [2,3]. Angiotensin receptor blockers (ARBs) are regarded as first-line therapy for hypertensive patients with T2DM. Moreover, a large-scale clinical trial showed that the use of ARBs prevented the onset of diabetes mellitus. Recently, ARBs received much attention in terms of decreasing oxidant stress and controlling inflammation in organs. Previously we reported that candesartan improved inflammatory parameters (HMW-ADN and PAI-1) in hypertensive patients with T2DM of long duration independently of blood pressure changes [4].

Pioglitazone is a diabetic medicine that improves insulin resistance. That pioglitazone directly reduces the incidence of cardiovascular events and stroke was shown by the PROactive Study, a large-scale clinical trial [5,6]. The protective action of pioglitazone on organs is thought to be through an anti-inflammatory action that reduces oxidant stress similar to ARBs as previously noted [7-10]. Therefore, using medicines such as pioglitazone in conjunction with an ARB might be useful not only with respect to blood pressure and plasma glucose control but also from the viewpoint of protection of organs.

Because the prevalence of metabolic syndrome has increased worldwide and the number of hypertensive patients with diabetes is also expected to increase, the opportunities for clinicians to use an ARB in conjunction with an anti-diabetic medication are expected to grow. Therefore, verifying the effectiveness of this combination therapy in patients with hypertension and diabetes is of clinical significance. However, the utility of such combination therapy from the viewpoint of their anti-inflammatory effects has not been clarified.

In this study, we administered as an ARB candesartan, which is the only sartan approved for use in chronic heart failure patients in Japan, and pioglitazone, an anti-diabetic medicine that improves insulin resistance, for 6 months to hypertensive patients with T2DM of long duration but without a history of cardiovascular events. We evaluated whether there was improvement in the serum inflammatory parameters of high-molecular-weight adiponectin (HMW-ADN), plasminogen activator inhibitor-1 (PAI-1), highly sensitive C-reactive protein (Hs-CRP), vascular cell adhesion molecule-1 (VCAM-1), and urinary-8-hydroxydeoxyguanosine (U-8-OHdG). We then analyzed the relationship between the degree of lowering of HbA1c and blood pressure and changes in inflammatory factors. Furthermore, the relationship between pulse pressure and the degree of lowering of HbA1c and changes in inflammatory factors was analyzed. Finally, we analyzed predictors of which patients would benefit from co-administration of candesartan with pioglitazone through their organ protective effects.

## Methods

### Participants

In this prospective study, patients were targeted for enrollment among hypertensive patients with T2DM (defined according to ADA criteria [11] or the use of anti-diabetic agents) who regularly attended the Jikei University School of Medicine affiliated hospital for treatment. We enrolled 41 patients (34 males and 7 females, 25–75 years old, average 60 years) who had hypertension (defined as diastolic blood pressure [DBP] ≧80 mmHg or systolic blood pressure [SBP] ≧130 mmHg, average 141/86) or who were taking anti-hypertensive agents (Table 1). Patients with secondary hypertension were excluded, as were patients with impaired liver function defined by plasma aminotransferase (or aspartate aminotransferase) >39 mUml (normal values: 11–39 mUml) and alanine aminotransferase >34 mUml (normal values: 11–34 mUml) or impaired kidney function (defined as serum creatinine level >1.3 mg/100 ml (normal values: 0.6–1.3 mg/100 ml). Also excluded were those with unstable cardiovascular conditions (e.g., New York Heart Association class I–IV congestive heart failure or a history of myocardial infarction or stroke) or who had a cerebrovascular incident within 6 months of study enrollment. Women who were pregnant, lactating or who might become pregnant due to inadequate contraceptive precautions were also excluded. Patients with known contraindications or intolerance to candesartan or pioglitazone were also excluded. Patients were simultaneously administered 12 mg candesartan and 15 mg pioglitazone daily for a duration of 6 months. If at the beginning of the study patients were taking an ARB or ACE-I, that drug was replaced with 12 mg candesartan. Anti-hypertensive and anti-diabetic agents were not changed during this study. The study protocol was approved by the institutional review board at Jikei University School of Medicine and conducted in accordance with the Declaration of Helsinki and its amendments. After a full explanation of the study, all patients gave written informed consent.

**Table 1:** Baseline data on study subjects

**N**		**41**	
Sex	(M/F)	34	(87.2%)
Age	(year)	59.74	(1.29)
BW	(kg)	73.24	(2.50)
BMI	(kg/m2)	26.40	(0.71)
Blood pressure	(SBP/DBP)(mmHg)	141(2.14)/85.5(1.57)	(1.57)
Duration	(year)	16.32	(1.67)
Laboratory data			
HbA1c	(%)	8.32	(1.23)
FPG	(mol/l)	9.72	(0.49)
LDL-C	(mg/dl)	108.8	(3.41)
HDL-C	(mg/dl)	48.8	(2.13)
Triglycerides	(mg/dl)	148.0	(11.72)
Creatinine	(mg/dl)	0.81	(0.04)
Blood glucose lowering treatment			
Insulin		1	
Insulin+OHA		3	
OHA		32	
Diet		3	

F, female; M, male, BMI, Body-mass index; DBP, diastolic blood pressure; SBP, systolic blood pressure; FPG, fasting plasma glucose; HbA1c, glycated hemoglobin; HDL-C, High density lipoprotein-cholesterol; LDL-C, Low density lipoprotein-cholesterol; OHA, oral hypoglycemic agent. Data are expressed as means (SEM) or n and %.

### Baseline assessment of participants

Before starting the study, all patients underwent an initial screening assessment that included a medical history and physical examination. We evaluated patients at the start of the study to establish baseline values, then again after the 6th month of treatment. Parameters examined were as follows: body weight, body mass index (BMI), SBP, DBP, HbA1c, fasting plasma glucose (FPG), HMW-ADN, PAI-1, Hs-CRP, VCAM-1, and U-8-OHdG. To evaluate tolerability of candesartan and pioglitazone, all adverse events were recorded. All plasmatic parameters were measured after a 12-h overnight fast. In all cases, venous blood samples were taken between 800 and 900 h. We used plasma obtained by the addition of Na2-EDTA (1 mgml_1) and centrifuged at 3000 g for 15 min at 4°C. All measurements were performed in a central laboratory. BMI was calculated as weight (kg) divided by the square of height (m). Height and weight were determined using a standard scale (SYSTEM 502, TANITA, Tokyo, Japan). Blood pressure measurements were obtained from the right arm while patients were in a seated position using a standard sphygmomanometer (ADVANCE BP-203RVIIIC/D, OMRON colin, Tokyo, Japan) (Korotkoff I and V) with an appropriately-sized cuff. Furthermore, the same investigator measured patients’ blood pressure at each visit, always in the morning and after the patient had rested for at least 10 min in a quiet room. Three successive blood pressure readings were obtained at 1-min intervals, and the mean of the three readings was calculated. HbA1c level was measured by high-performance liquid chromatography (HPLC) (HLC723-G9, TOSOH, Tokyo, Japan), with intra- and interassay coefficients of variation (CsV) of 1%. Plasma glucose was assayed by the glucose-oxidase method (GA08II, A&T, Yokohama, Japan) with intra- and inter-assay CsV of 0.8%. Plasma HMW-ADN level was determined using a chemiluminescent enzyme immunoassay (CLEIA) (Fuji Rebio, Tokyo, Japan). Plasma VCAM-1 level (normal values 277–836 ng/ml) was determined using an enzyme-linked immunosorbent assay (ELISA) (R&D Systems, Inc., Minneapolis, MN, USA). Plasma PAI-1 levels (normal values ≤50 ng/ml) were determined using a latex photometric immunoassay (LPIA) (Mitsubishi Chemical Medience, Tokyo, Japan). The U-8-OHdG level (normal values l: 6.1-16.3 ng/mg·cr) was measured using HPLC (Mitsubishi Chemical Medience, Tokyo, Japan). Plasma Hs-CRP level (normal values <0.3 mg/dl) was measured using the latex agglutination nephelometry method (Siemens Healthcare Diagnostics, Inc., Marburg, Germany).

### Statistical analysis

Statistical analysis of data was performed using the Statistical Package for Social Sciences software, version 19.0 (SPSS, Chicago, IL, USA). Data are presented as the mean ± s.e. For all statistical analyses, P<0.05 was considered statistically significant.

## Results

### Characteristics of study sample

**Figure 1:**
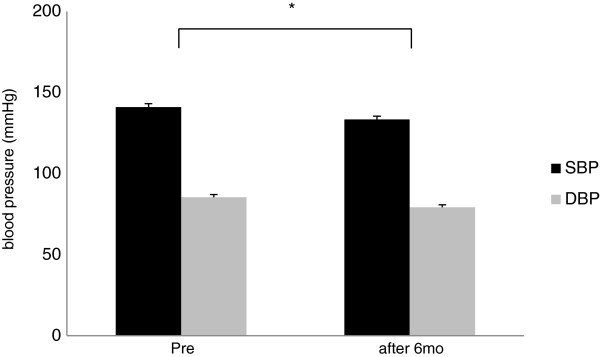
**Changes from baseline of blood pressure parameters after treatment.** Systolic blood pressure (SBP) and diastolic blood pressure (DPB). *P<0.01 vs. Pre. Error bars indicate SEM for SBP and for DBP.

**Figure 2:**
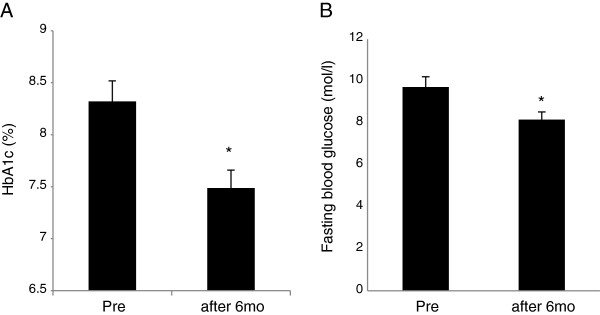
**Changes from baseline of glucose parameters after treatment.** HbA1c (**A**). and fasting plasma glucose (FPG); (**B**). *P<0.01 vs. Pre. Error bars indicate SEM for HbA1c and for FPG.

Of the 41 patients who were enrolled in the study, 39 completed the study. The reason for premature withdrawal was lost-to-follow-up. Characteristics of the patient population upon entering the study and the antidiabetic treatments taken before and during the study are shown in Table 1. Patient data at baseline and after the 6th month of the study are shown in Table 2. Significant improvements from baseline values were observed in both SBP and DBP after 6 months *P<0.01, Table 2 and Figure 1). HbA1c and FPG values were significantly improved from baseline after the 6-month treatment period *P<0.01, Table 2 and Figure 2).

**Table 2:** Patient data at baseline and after 6-mo study period

**N**		**41**		**39**	
Dropped out		N/A		2	
BW(kg)		73.24	(2.50)	74.8	(2.29
BMI(kg/m2)		26.40	(0.71)	26.95	(0.69)
Blood pressure(SBP/DBP)(mmHg)		141	(2.14)/	133	(2.04)*/
		85.5	(1.57)	79.3	(1.51)
Laboratory data					
HbA1c	(%)	8.32	(0.20)	7.48	(0.17)*
FPG	(mol/l)	9.72	(0.49)	8.17	(0.37)*
HMW-ADN	(μg/ml)	4.50	(0.60)	7.99	(0.71)*
PAl-1	(mg/dl)	32.7	(2.60)	24.1	(1.89)*
VCAM-1	(mg/dl)	698.9	(27.9)	680.6	(30.15)
U-8-OHdg	(ng/mg cr)	11.7	(0.62)	11.7	(0.44)
Hs-CRP	(mg/dl)	0.27	(0.05)	0.19	(0.04)

BMI, Body-mass index; DBP, diastolic blood pressure; SBP, systolic blood pressure; FPG, fasting plasma glucose; HbA1c, glycated hemoglobin; HMW-ADN, high molecular weight-adiponectin; Hs-CRP, high sensitivity-Creactive protein; PAI-1, plasminogen activator inhibitor-1; U-8-OHdg, urinary-8-hydroxydeoxygnosine; VCAM-1, vascular cell adhesion molecule-1. Data are expressed as means (sem). or n and %. *P<0.01.

**Figure 3:**
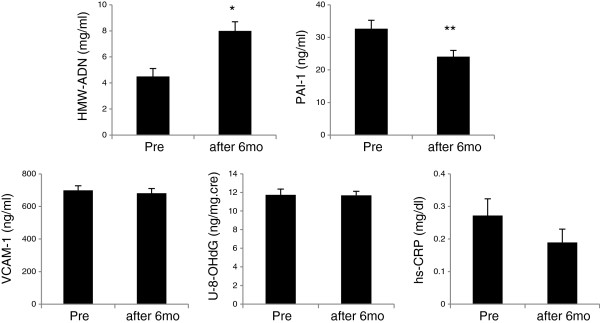
**Changes from baseline of inflammatory parameters after treatment.** HMW-ADN, high molecular weight adiponectin; Hs-CRP, highly sensitive C-reactive protein; PAI-1, plasminogen activator inhibitor-1; U-8-OHdG, urinary 8-hydroxydeoxyguanosine; VCAM-1, vascular cell adhesion molecule-1. Error bars indicate SEM for HMW-AND. *P<0.05 vs Pre. **P<0.01 *P=0.002 vs Pre. Error bars indicate SEM for PAI-1.

Furthermore, significant improvements in baseline values for HMW-ADN and PAI-1 were recorded in all patients after 6 months of treatment (*P<0.05, **P<0.01, respectively). VCAM-1, U-8-OHdG, and Hs-CRP values did not decrease from baseline after 6 months of candesartan with pioglitazone treatment (Figure 3).

### Correlations

**Figure 4:**
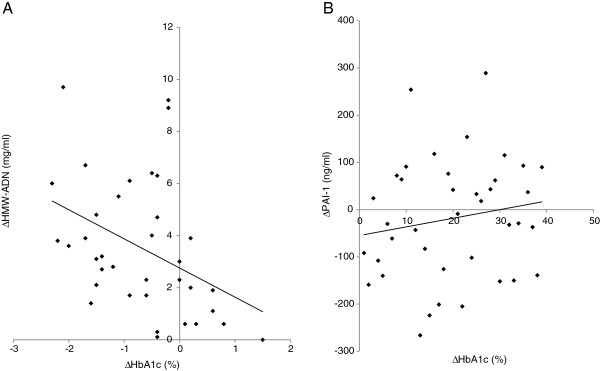
**Metabolic and inflammatory factors vs. ⊿HbA1c.** (**A**). ⊿HMW-ADN vs. ⊿HbA1c: r=−0.382, P=0.018; (**B**). ⊿PAI-1 vs. ⊿HbA1c: r=0.404, P=0.015.

Stepwise multilinear regression analysis was performed to establish which factors could best predict changes in patients’ inflammatory factors. Co-administration of candesartan with pioglitazone improved inflammatory parameters (HMW-ADN and PAI-1) in hypertensive patients with T2DM of long duration independent of blood pressure changes. In addition, the following positive correlations with changes in HbA1c (⊿HbA1c) were revealed: ⊿HbA1c vs. ⊿HMW-ADN (r=−0.382, P=0.018, Figure 4A) and ⊿HbA1c vs. ⊿PAI-1(r=0.404, P=0.015, Figure 4B). Results of other correlation analyses did not show patterns of associations with regard to ⊿HbA1c with ⊿VCAM-1, ⊿8-OHdG, and ⊿Hs-CRP (Additional file 1: Figure S1).

**Figure 5:**
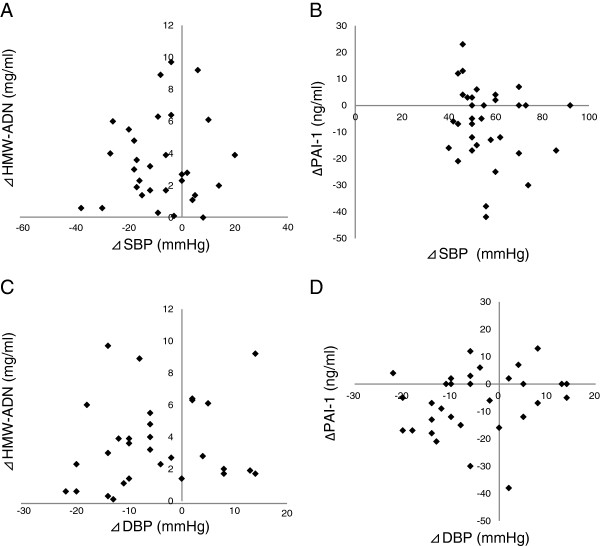
**Metabolic and inflammatory factors vs. ⊿SBP and ⊿DBP.** (**A**). ⊿HMW-ADN vs. ⊿SBP: r=−0.090, P=0.630; (**B**). ⊿PAI-1 vs. ⊿SBP: r=0.241, P=0.207; (**C**). ⊿HMW-ADN vs. ⊿DBP: r=−0.382, P=0.165; (**D**). ⊿PAI-1 vs. ⊿DBP: r=0.210, P=0.285.

**Figure 6:**
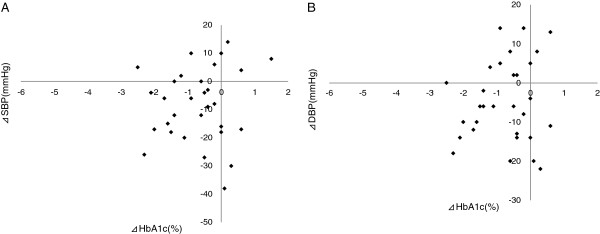
**Blood pressure vs. ⊿HbA1c.** (**A**). ⊿SBP: r= 0.151, P=0.410; (**B**). ⊿DBP: r=0.124, P=0.505.

There were no correlations between changes in SBP and DBP (⊿SBP and ⊿DBP) and the following parameters: ⊿SBP vs. ⊿HMW-ADN, ⊿SBP vs. ⊿PAI-1, ⊿DBP vs. ⊿HMW-ADN, and ⊿DBP vs. ⊿PAI-1 (Figure 5A, B, C, and D, respectively). And there were no correlations between ⊿SBP, ⊿DBP and the following parameters: ⊿SBP vs. ⊿VCAM-1, ⊿SBP vs. ⊿U-8-OHdG, ⊿SBP vs. ⊿Hs-CRP, ⊿DBP vs. ⊿VCAM-1, ⊿DBP vs. ⊿U-8-OHdG, ⊿DBP vs. ⊿Hs-CRP (Additional file 2: Figure S2, Additional file 3: Figure S3). Furthermore, correlation analyses indicated that no associations existed between ⊿SBP and ⊿DBP and ⊿HbA1c (Figure 6A, B).

## Discussion

In this prospective trial of patients with hypertension and T2DM, we observed that co-administration of candesartan with pioglitazone had beneficial effects with regard to hypertension, inflammation, and adipose tissue metabolism. In particular, patients receiving treatment with both agents experienced a significant improvement in blood pressure, HbA1c, HMW-ADN, and PAI-1. Furthermore, we analyzed predictive factors in patients who received the greatest benefits from the co-administration of candesartan with pioglitazone.

### Co-administration of candesartan with pioglitazone

Both candesartan and pioglitazone were clinically proven to provide direct cardiovascular protection through pleiotropic effects [12-16] not only in basic studies. Furthermore, the anti-inflammatory effect and suppression of oxidative stress in tissues have been the center of attention as the pleiotropic action of both drugs. The combination of candesartan and pioglitazone was more effective than candesartan monotherapy in pigs and rats with cardiovascular risks [17,18]. Pioglitazone and candesartan in combination were demonstrated to present additive protective effects on renal fibrosis in an experimental mouse model, suggesting that their use in combination would be an effective treatment for chronic kidney disease [19]. Moreover, in patients with T2DM in whom prior hypoglycemic therapies failed, pioglitazone co-administration improved blood lipid and glycemic profiles, thus decreasing the proportion of patients with a high microvascular or macrovascular risk [20]. Therefore, we retrospectively analyzed the effects of co-administration therapy compared to the use of candesartan monotherapy on HMW-ADN and PAI-1, which we addressed previously [4]. After adjustment for age, BMI, HbA1c and blood pressure values, the co-administration arm compared to the candesartan administration only arm had a significant improvement in ⊿HMW-ADN and a tendency toward improvement in ⊿PAI-1 (%) (Additional file 4: Figure S4).

That there has been an increase in hypertensive patients with T2DM supports the clinical importance of this research. Our data suggest that this therapy was effective from the viewpoint of inflammatory parameters (HMW-ADN and PAI-1) for hypertensive patients with T2DM with a cardiovascular risk. However, these data were not investigated at the same time for the same purpose, which might be considered a limitation of this study. Large-scale prospective multi-arm studies are expected in the future.

### Correlation between ⊿HbA1c and inflammatory factors ⊿HMW-ADN and ⊿PAI-1

A positive correlation was observed in ⊿HMW-ADN and ⊿PAI-1 and changes in HbA1c (Figure 4A, B). Our data might be thought to indicate that the improvement in HMW-ADN·PAI-1 with plasma glucose (HbA1c) created a virtuous circle. Did the improvement in plasma glucose cause the improvement in inflammatory parameters or was the opposite correct? The truth is unknown. But our results showed close relationships between inflammation and blood glucose levels. Pioglitazone has a dose–response effect on insulin sensitivity and insulin secretion in T2DM [21-24]. We are inclined to the opinion that if these patients would take pioglitazone 30–45 mg daily, further improvement in the inflammatory parameters in addition to HbA1c would be achieved by a virtuous circle.

### Correlation between ⊿SBP,⊿DBP and inflammatory factors ⊿HMW-ADN and ⊿PAI-1

⊿SBP and ⊿DBP correlation analyses did not indicate patterns of associations with these parameters (Figure 5A, B, C, D). These results might indicate that candesartan improved these parameters directly and not through changes in blood pressure or that it had lesser effects in patients with diabetes mellitus of a long duration.

### Correlation between ⊿HbA1c and pulse pressure values at baseline

**Figure 7:**
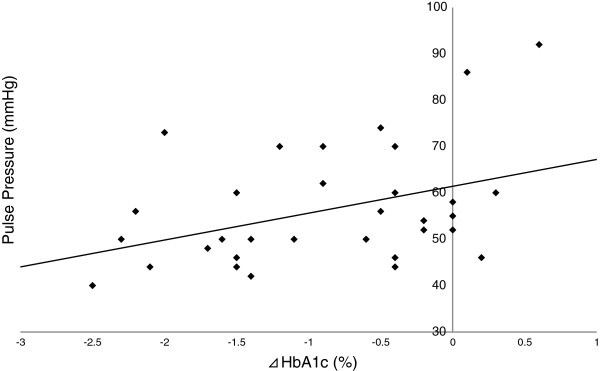
**Single correlation analysis of pulse pressure and degree of lowering of HbA1c.** r=0.370, P=0.034.

Interestingly, by correlation analysis we observed a significant correlation between the degree of lowering of HbA1c (⊿HbA1c) and pulse pressure values at baseline. Furthermore, multiple regression analysis showed that pulse pressure was independent of age and BMI (Table 3 and Figure 7). In summary, these data suggest that pulse pressure values at baseline can become a key predictive factor of changes in patients’ HbA1c. There is evidence that pulse pressure is an index parameter of arteriosclerosis [25,26]. In brief, our data suggest that the patients in our study who did not have advanced atherosclerosis were those who could benefit most from this treatment. These results are unique. We think that there is an important relationship between blood pressure and plasma glucose, and the reason for this should be examined in the future.

**Table 3:** Multivariate regression analysis of variables for ⊿HbA1c

	**β**	**P value**
Pulse pressure	0.492	0.012*
Age	0.178	0.298(NS)
Body mass index	-0.084	0.653(NS)

### Benefits and risks of pioglitazone

Among our study patients, we did not observe increases in body weight, exacerbation of heart failure, and the development of bladder cancer after 6 months of treatment. In France, analysis revealed a significant association between pioglitazone and bladder cancer in men [27]. But in Asia, two reports showed no relation of thiazolidinediones with bladder cancer. [28,29]. Cariou et al. noted that the thiazolidinediones, mainly pioglitazone, remain effective and useful anti-diabetic drugs with a unique insulin-sensitizing action. This therapeutic option must be validated by a decrease in HbA1c and the lack of serious adverse events [30]. Also, with regard to cardiovascular outcomes, pioglitazone is useful for patients with cardiovascular and microvascular risks [12,13,20]. Therefore, pioglitazone has benefits in hypertensive patients with T2DM who are at high risk for cardiovascular disease.

### Study limitations

This was a pilot study. It was a single group pre-post study of a small number of patients with unbalanced distribution between men and women. Co-administration therapy for 6 months was less effective with regard to VCAM-1, U-8-OHdG, and Hs-CRP. The precise reason is unknown. Results of larger clinical trials evaluating the anti-inflammatory effects of the co-administration of candesartan with pioglitazone are awaited.

## Conclusion

In conclusion, the simultaneous co-administration of candesartan with pioglitazone improved inflammatory parameters (HMW-ADN and PAI-1), and improvements in inflammatory parameters were correlated with ⊿HbA1c. Pulse pressure values at baseline can become a key predictive factor for changes in patients’ HbA1c. The co-administration of candesartan with pioglitazone could be an effective therapeutic strategy for treating hypertensive patients with T2DM.

## Abbreviations

BMI: Body mass index; DPB: Diastolic blood pressure; ELISA: Enzyme-linked immunosorbent assay; FPG: Fasting plasma glucose; HbA1c: Glycated hemoglobin A1c; HMW-ADN: High-molecular-weight adiponectin; Hs-CRP: Highly sensitive C-reactive protein; PAI-1: Plasminogen activator inhibitor-1; SBP: Systolic blood pressure; T2DM: Type 2 diabetes mellitus; U-8-OHdG: Urinary 8-hydroxydeoxyguanosine; VCAM-1: Vascular cell adhesion molecule-1.

## Competing interests

The authors declare that they have no competing interests.

## Authors’ contributions

HS and MS conceptualized the research hypothesis and analyses, researched the data, performed all of the statistical analyses and wrote the manuscript. TH, HI, TI, KO, NS and YK reviewed and edited the manuscript. KT, MY and KU assisted in conceptualizing the research question and reviewed and edited the manuscript. All authors read and approved the final manuscript.

## Supplementary Material

Additional file 1: Figure S1 Inflammatory factors vs. ⊿HbA1c. (A). ⊿VCAM-1 vs. ⊿HbA1c: r=0.318, P=0.058; (B). ⊿8-OHdG vs. ⊿HbA1c: r= 0.215, P=.201; (C). ⊿Hs-CRP vs. ⊿HbA1c; r= 0.239; P=0.203. Click here for file

Additional file 2: Figure S2 Inflammatory factors vs. ⊿SBP. (A). ⊿VCAM-1 vs. ⊿SBP: r=−0.085, P=0.642; (B). ⊿U-8-OHdG vs. ⊿SBP: r=0.043, P=0.823; (C). ⊿Hs-CRP vs. ⊿SBP: r=−0.278, P=0.170. Click here for file

Additional file 3: Figure S3 Inflammatory factors vs. ⊿DBP. (A). ⊿VCAM-1 vs. ⊿DBP: r=−0.066, P=0.726; (B). ⊿U-8-OHdG vs. ⊿DBP: r=−0.132, P=0.494; (C). ⊿Hs-CRP vs. ⊿DBP: r=−0.286, P=0.156. Click here for file

Additional file 4: Figure S4 Co-administration vs. single candesartan. After adjusted HbA1c in both patients, co-administration arm compared to candesartan administration only arm significantly improved in ⊿HMW-ADN and ⊿PAI-1 (%). *P<0.05 vs Candesartan (Can). Error bars indicate SEM for Can and for Candesartan + Pioglitazone (Can + Pio). Click here for file
