# Supplementary material for: Effects of co-administration of candesartan with pioglitazone on inflammatory parameters in hypertensive patients with type 2 diabetes mellitus: a preliminary report
Source: Cardiovasc Diabetol. 2013 May 2;12:71. doi: 10.1186/1475-2840-12-71 (PMC3663745; doi:10.1186/1475-2840-12-71)
Supplement: Additional file 1: Figure S1 — Inflammatory factors vs. ⊿HbA1c. (A). ⊿VCAM-1 vs. ⊿HbA1c: r=0.318, P=0.058; (B). ⊿8-OHdG vs. ⊿HbA1c: r= 0.215, P=.201; (C). ⊿Hs-CRP vs. ⊿HbA1c; r= 0.239; P=0.203. [file 1475-2840-12-71-S1.pptx]

## Slide 1
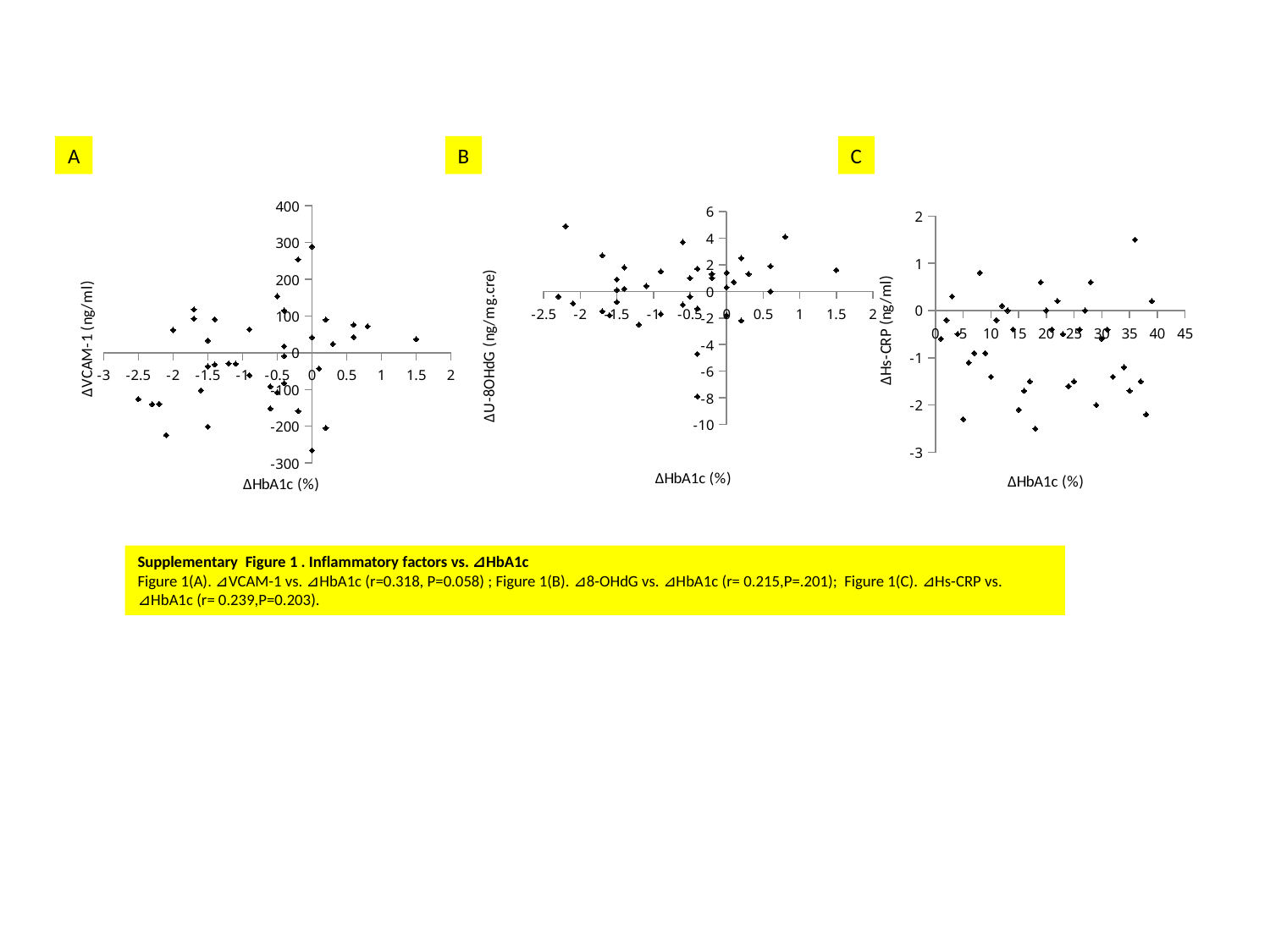

A
B
C
### Chart
| Category | | | |
|---|---|---|---|
### Chart
| Category | |
|---|---|
### Chart
| Category | |
|---|---|Supplementary Figure 1 . Inflammatory factors vs. ⊿HbA1c
Figure 1(A). ⊿VCAM-1 vs. ⊿HbA1c (r=0.318, P=0.058) ; Figure 1(B). ⊿8-OHdG vs. ⊿HbA1c (r= 0.215,P=.201); Figure 1(C). ⊿Hs-CRP vs. ⊿HbA1c (r= 0.239,P=0.203).
