# Supplementary material for: Effects of co-administration of candesartan with pioglitazone on inflammatory parameters in hypertensive patients with type 2 diabetes mellitus: a preliminary report
Source: Cardiovasc Diabetol. 2013 May 2;12:71. doi: 10.1186/1475-2840-12-71 (PMC3663745; doi:10.1186/1475-2840-12-71)
Supplement: Additional file 2: Figure S2 — Inflammatory factors vs. ⊿SBP. (A). ⊿VCAM-1 vs. ⊿SBP: r=−0.085, P=0.642; (B). ⊿U-8-OHdG vs. ⊿SBP: r=0.043, P=0.823; (C). ⊿Hs-CRP vs. ⊿SBP: r=−0.278, P=0.170. [file 1475-2840-12-71-S2.pptx]

## Slide 1
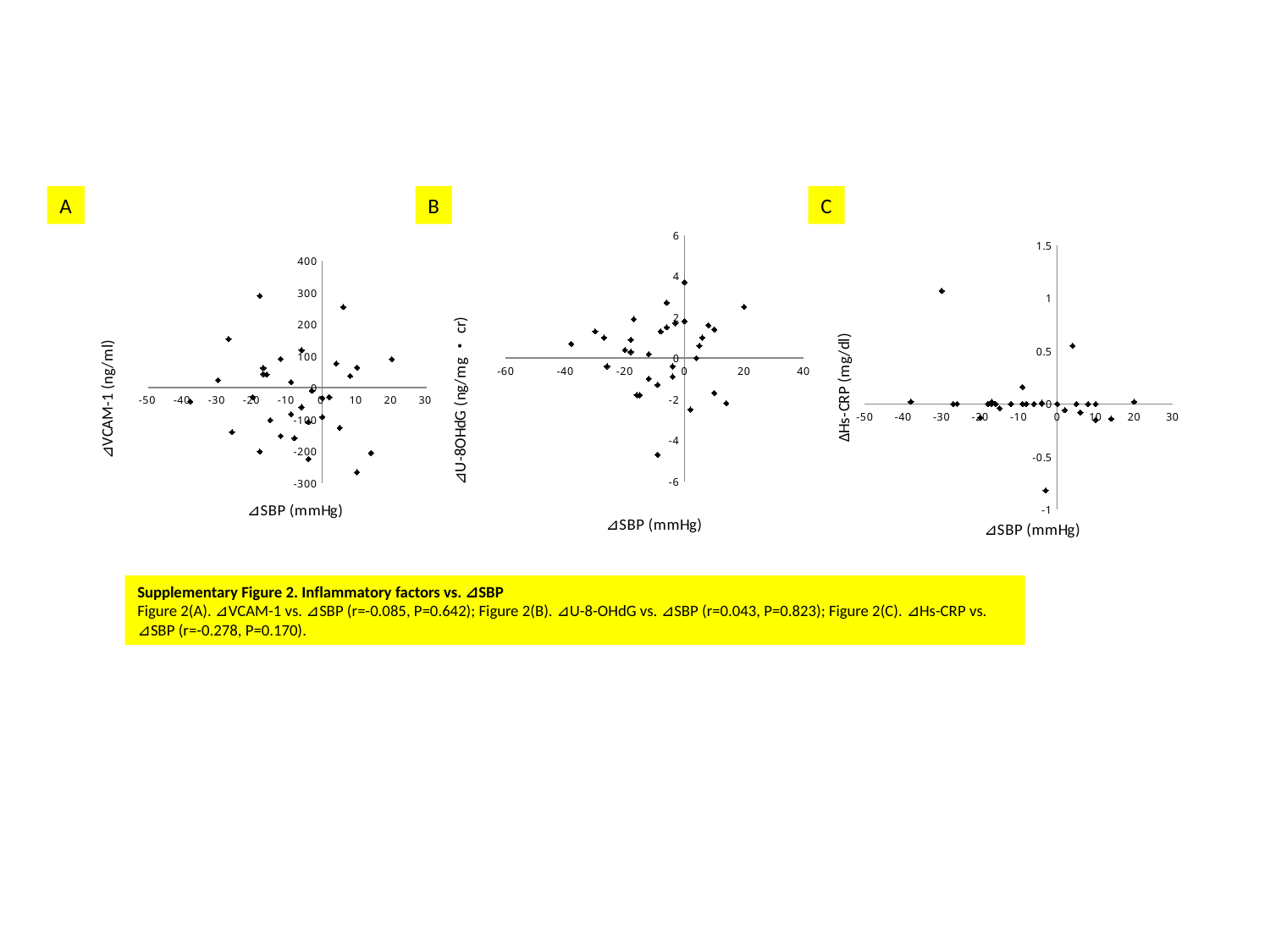

A
B
C
### Chart
| Category | |
|---|---|
### Chart
| Category | |
|---|---|
### Chart
| Category | |
|---|---|Supplementary Figure 2. Inflammatory factors vs. ⊿SBP
Figure 2(A). ⊿VCAM-1 vs. ⊿SBP (r=-0.085, P=0.642); Figure 2(B). ⊿U-8-OHdG vs. ⊿SBP (r=0.043, P=0.823); Figure 2(C). ⊿Hs-CRP vs. ⊿SBP (r=-0.278, P=0.170).
