# Supplementary material for: Effects of co-administration of candesartan with pioglitazone on inflammatory parameters in hypertensive patients with type 2 diabetes mellitus: a preliminary report
Source: Cardiovasc Diabetol. 2013 May 2;12:71. doi: 10.1186/1475-2840-12-71 (PMC3663745; doi:10.1186/1475-2840-12-71)
Supplement: Additional file 3: Figure S3 — Inflammatory factors vs. ⊿DBP. (A). ⊿VCAM-1 vs. ⊿DBP: r=−0.066, P=0.726; (B). ⊿U-8-OHdG vs. ⊿DBP: r=−0.132, P=0.494; (C). ⊿Hs-CRP vs. ⊿DBP: r=−0.286, P=0.156. [file 1475-2840-12-71-S3.pptx]

## Slide 1
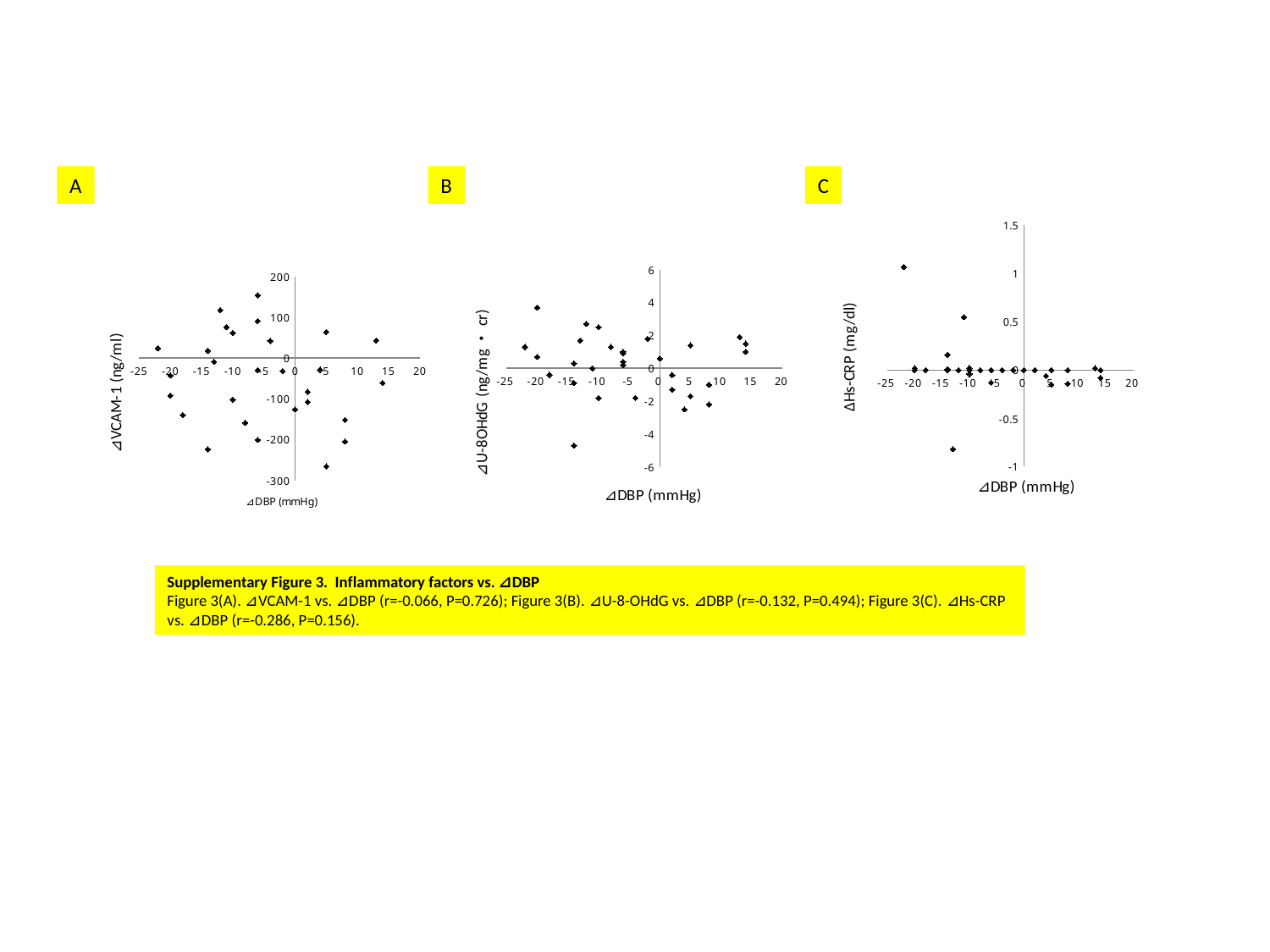

A
B
C
### Chart
| Category | |
|---|---|
### Chart
| Category | |
|---|---|
### Chart
| Category | |
|---|---|Supplementary Figure 3. Inflammatory factors vs. ⊿DBP
Figure 3(A). ⊿VCAM-1 vs. ⊿DBP (r=-0.066, P=0.726); Figure 3(B). ⊿U-8-OHdG vs. ⊿DBP (r=-0.132, P=0.494); Figure 3(C). ⊿Hs-CRP vs. ⊿DBP (r=-0.286, P=0.156).
