# Supplementary material for: Effects of co-administration of candesartan with pioglitazone on inflammatory parameters in hypertensive patients with type 2 diabetes mellitus: a preliminary report
Source: Cardiovasc Diabetol. 2013 May 2;12:71. doi: 10.1186/1475-2840-12-71 (PMC3663745; doi:10.1186/1475-2840-12-71)
Supplement: Additional file 4: Figure S4 — Co-administration vs. single candesartan. After adjusted HbA1c in both patients, co-administration arm compared to candesartan administration only arm significantly improved in ⊿HMW-ADN and ⊿PAI-1 (%). *P<0.05 vs Candesartan (Can). Error bars indicate SEM for Can and for Candesartan + Pioglitazone (Can + Pio). [file 1475-2840-12-71-S4.pptx]

## Slide 1
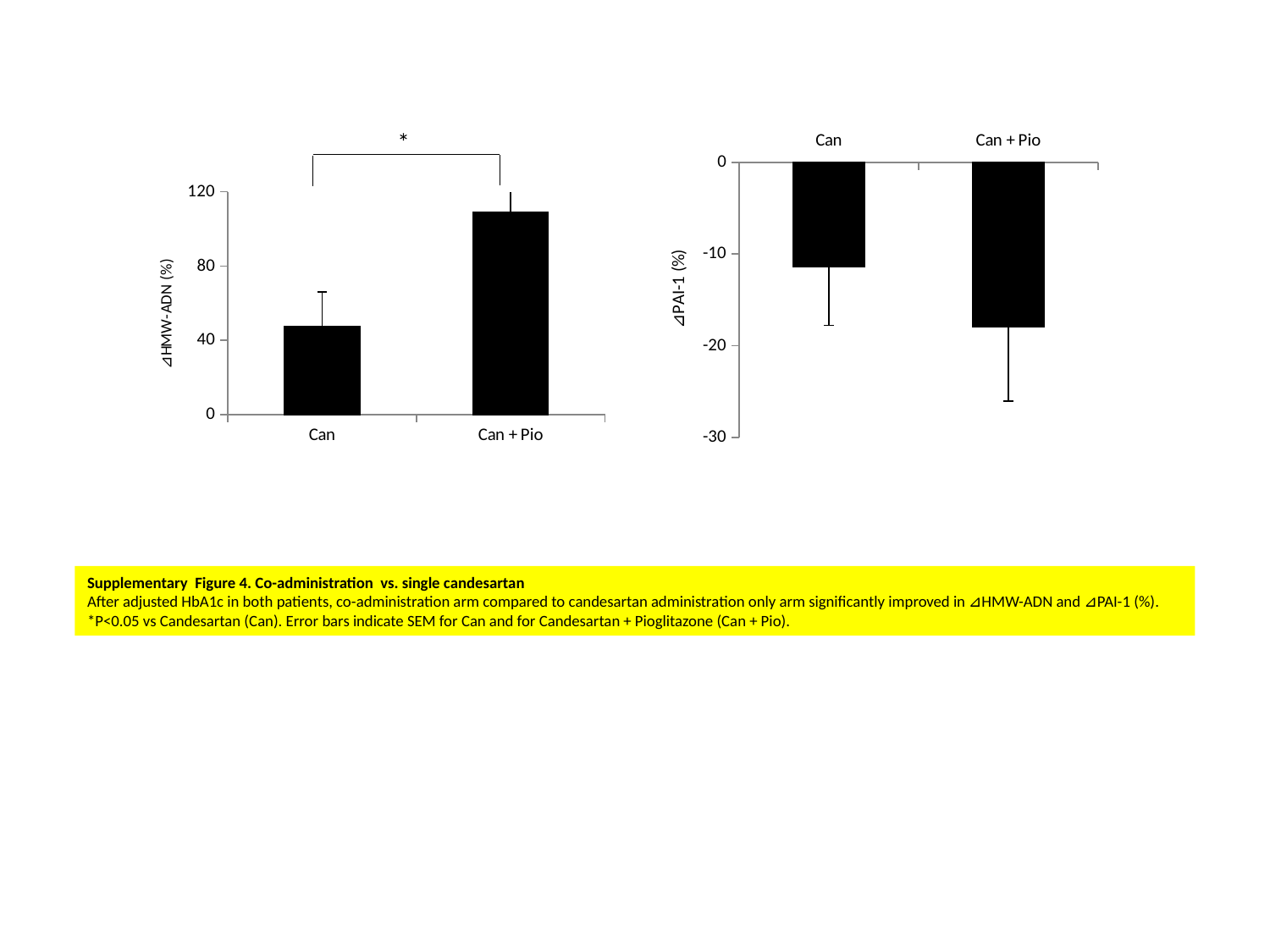

### Chart
| Category | |
|---|---|
| Can | 47.71785334917493 |
| Can + Pio | 109.26007780180318 |
### Chart
| Category | |
|---|---|
| Can | -11.445960049856811 |
| Can + Pio | -17.988879624048323 |Supplementary Figure 4. Co-administration vs. single candesartan
After adjusted HbA1c in both patients, co-administration arm compared to candesartan administration only arm significantly improved in ⊿HMW-ADN and ⊿PAI-1 (%). *P<0.05 vs Candesartan (Can). Error bars indicate SEM for Can and for Candesartan + Pioglitazone (Can + Pio).
